# Effect of cold atmospheric microwave plasma (CAMP) on wound healing in canine keratinocytes

**DOI:** 10.3389/fcell.2023.1105692

**Published:** 2023-01-25

**Authors:** Pattawika Lertpatipanpong, Chanin Sillapachaiyaporn, Garam Oh, Yeong-Hun Kang, Cheol-Yong Hwang, Seung Joon Baek

**Affiliations:** ^1^ Laboratory of Signal Transduction, Research Institute for Veterinary Science, College of Veterinary Medicine, Seoul National University, Seoul, South Korea; ^2^ Program in Clinical Biochemistry and Molecular Medicine, Department of Clinical Chemistry, Faculty of Allied Health Sciences, Chulalongkorn University, Bangkok, Thailand; ^3^ Laboratory of Veterinary Dermatology, Research Institute for Veterinary Science, College of Veterinary Medicine, Seoul National University, Seoul, South Korea

**Keywords:** wound healing, keratinocyte, cold plasma (CP), microwave plasma, RNA-Seq

## Abstract

Cutaneous wound healing is a biological process that occurs upon skin injury and involves different mechanisms to repair tissue damage. Improper healing or prolonged curation period of wound lesions may induce unpleasant complications. Cold atmospheric microwave plasma (CAMP) is an upcoming medical therapeutic option for skin infection and wound treatment. However, the molecular mechanisms of CAMP-mediated canine wound healing are not well characterized. Wound-healing activity was examined to elucidate the biological effects and molecular mechanisms of CAMP. Canine keratinocytes (CPEKs) were treated using CAMP, and their wound-healing activities were evaluated. The molecular mechanisms of that effect were examined, based on RNA-Seq analysis data, and verified using immunoblotting and polymerase chain reaction. It was found that the CAMP-treated cells exhibited a significant increase in cell migration evaluated by scratch assay in human keratinocytes (HaCaT) and canine keratinocytes (CPEK). Additionally, CAMP-treated CPEK cells showed a significant positive effect on cell invasion. The RNA-Seq data revealed that CAMP alters different genes and pathways in CPEK cells. Gene expression involved in the cell cycle, cell proliferation, angiogenesis, cell adhesion, and wound healing was upregulated in CAMP-treated cells compared with gas-activated media used as a control. The Hippo pathway was also analyzed, and the protein and mRNA levels of YAP were significantly increased in CAMP-treated cells. CAMP-treated CPEK cells indicated the downregulation of E-cadherin and upregulation of vimentin, Snail, and Slug at transcription and translation levels, contributing to a favorable effect on cell migration. Our findings suggested that CAMP treatment provided beneficial effects on the curative wound process through the induction of genes involved in wound healing, promotion of EMT, and increase in the molecular targets in the Hippo signaling pathway.

## 1 Introduction

The skin has the largest surface area among organs in the body and plays a role as a protective barrier to protect internal tissues against mechanical damage, microbial infection, ultraviolet radiation, and extreme temperatures (Rodrigues et al., 2019). Upon tissue injury, cutaneous wound healing remarkably takes part in the repair and regeneration of damaged cells. Therefore, wound healing is an important physiobiological process to maintain skin barrier and homeostasis, which comprises the multi-complexes of cellular processes. In mammals, the three main distinct stages upon cell injury are inflammation, tissue formation, and remodeling (Gurtner et al., 2008). The first stage of wound healing occurs immediately after tissue damage including inflammatory cascade reaction, immune system activation, and blood coagulation. The second stage is tissue formation characterized by cellular proliferation and migration of various cell types, including the migration of keratinocytes to the wound dermis, and the formation of a new blood vessel. Later, fibroblasts are activated, and some of those are differentiated into myofibroblasts, subsequently contributing to the accumulation of connective tissue, mainly collagen, which finally forms the scar (Opalenik and Davidson, 2005; Werner et al., 2007). At the latest stage, the remodeling of all previously activated processes is diminished, and the apoptosis of macrophages, myofibroblasts, and endothelial cells occurs (Gurtner et al., 2008). Skin damage is a common and typical event in veterinary patients, including dogs. Although wounds can be healed by biological functions in the body, the delay in lesion repair may lead to complicating conditions. Therefore, wound management is the critical step to confronting undesired circumstances.

The application of plasma as a new therapeutic option in animal clinics has been increasing, although there has been lacking evidence about its molecular mechanism. In cold atmospheric microwave plasma (CAMP), cooling of ions and uncharged molecules is effective while the gases are still at a low temperature (Fridman et al., 2008), whereas the thermal microwave plasma produces lots of heat and high energy which may be harmful to living cells. There are many suggestions for the clinical application of cold plasma in the medical field, such as anti-flora bacteria on the skin (Wende et al., 2010), tissue regeneration (Barton et al., 2013; VON Woedtke et al., 2019), chronic wound healing (Isbary et al., 2010; Isbary et al., 2012; Assadian et al., 2019; VON Woedtke et al., 2019), and cancer treatment (Keidar, 2015; Gay-Mimbrera et al., 2016). Such therapeutic outcomes may be caused by its ionized energy and biologically active components, produced by gases such as argon or helium flowing through an electric field. Plasma can produce a set of photons, reactive oxygen species (ROS), reactive nitrogen oxide species (RNOS), and UV radiation, which can exert synergistic biological effects (Judée et al., 2014; Graves, 2017). In addition, CAMP treatment was well-tolerated and did not show any concerned adverse side effects in humans (Heinlin et al., 2013) and dogs (Lee et al., 2022). Therefore, CAMP treatment is safe and may be a potential clinical therapeutic option for skin diseases. Additionally, CAMP therapy is a safe approach to treating skin and employs an easy-to-access device, thus contributing to promising dermatologic applications of CAMP.

The Hippo pathway is one of the well-known pathways involved in organ development, epithelial homeostasis, tissue regeneration, wound healing, and immune modulation. This pathway contains multiplex signals that culminate to direct the function of the transcriptional regulators YAP and TAZ (orthologues of Yorkie in Drosophila) (Dey et al., 2020). The Hippo signaling pathway initiates with MST1/MST2 activation in extracellular stimulation response and contributes to the phosphorylation of SAV1, MOB1A/MOB1B, LATS1/2, and YAP/TAZ, resulting in YAP/TAZ degradation. In contrast, the hippo signaling inactivation leads to YAP/TAZ dephosphorylation and translocation to the nucleus, where they bind to the transcriptional enhancer factor (TEA)-domain (TEAD) family transcription factors and regulate their target gene expression to control various biological functions (Elbediwy et al., 2016). The crucial YAP/TAZ target genes that are related to cell growth, proliferation, and migration include CTGF (also known as CCN2), CYR61, MYC, AXL, BIRC5, and CCND1 (Kalluri and Weinberg, 2009). YAP/TAZ plays a role in basal stem/progenitor cell control, as a cell proliferation and tissue renewal regulator, in apical and basal epidermis. The elevation of nuclear YAP and TAZ localization is observed in the basal layer of skin, and these nuclear levels are elevated upon wound healing or tumor formation (Lei et al., 2008). In addition, epithelial–mesenchymal transition (EMT), a process in which epithelial cells are deprived of their phenotypes of cell polarity and cell–cell adhesion, and promote mesenchymal cell properties, resulting in the gain of migratory capacity, is one of the keys for successful wound repair. There are three types of EMT, and the type 2 EMT is associated with wound healing, tissue regeneration, and organ fibrosis (Pan, 2010). Considerable studies have shown that YAP/TAZ is a key regulator of the epithelial–mesenchymal transition (EMT) process. These results imply that the overexpression of YAP/TAZ induces EMT, whereas the inhibition of YAP/TAZ reduces EMT (Zhang et al., 2009; Yamaguchi and Taouk, 2020). Thus, the regulation of the Hippo pathway not only directly regulates wound healing on its own but also indirectly controls the EMT process, leading to augmentation of wound-repairing capacity.

The combination of advanced veterinary care and intensive research and pet owner demands has led to concern about the lifespan of those animals. For enhanced clinical outcomes in veterinary wound care, there has been a notable rise in commercially available inventions for use in companion animals, including topical agents, dressings, biologics, closure devices, and negative pressure wound therapy. In addition, dog models have been used as translational models for both veterinary and human applications to investigate the efficacy of various wound care therapies (Volk and Bohling, 2013). This study was designed to examine the potential molecular mechanisms of the wound-healing activity induced by CAMP in canine keratinocytes using RNA-Seq and *in vitro* experiments. Our results indicated that CAMP provides an advantageous effect on wound care by regulating the genes involved in wound healing, EMT, and Hippo signaling pathway.

## 2 Materials and methods

### 2.1 Plasma machine

**FIGURE 1:**
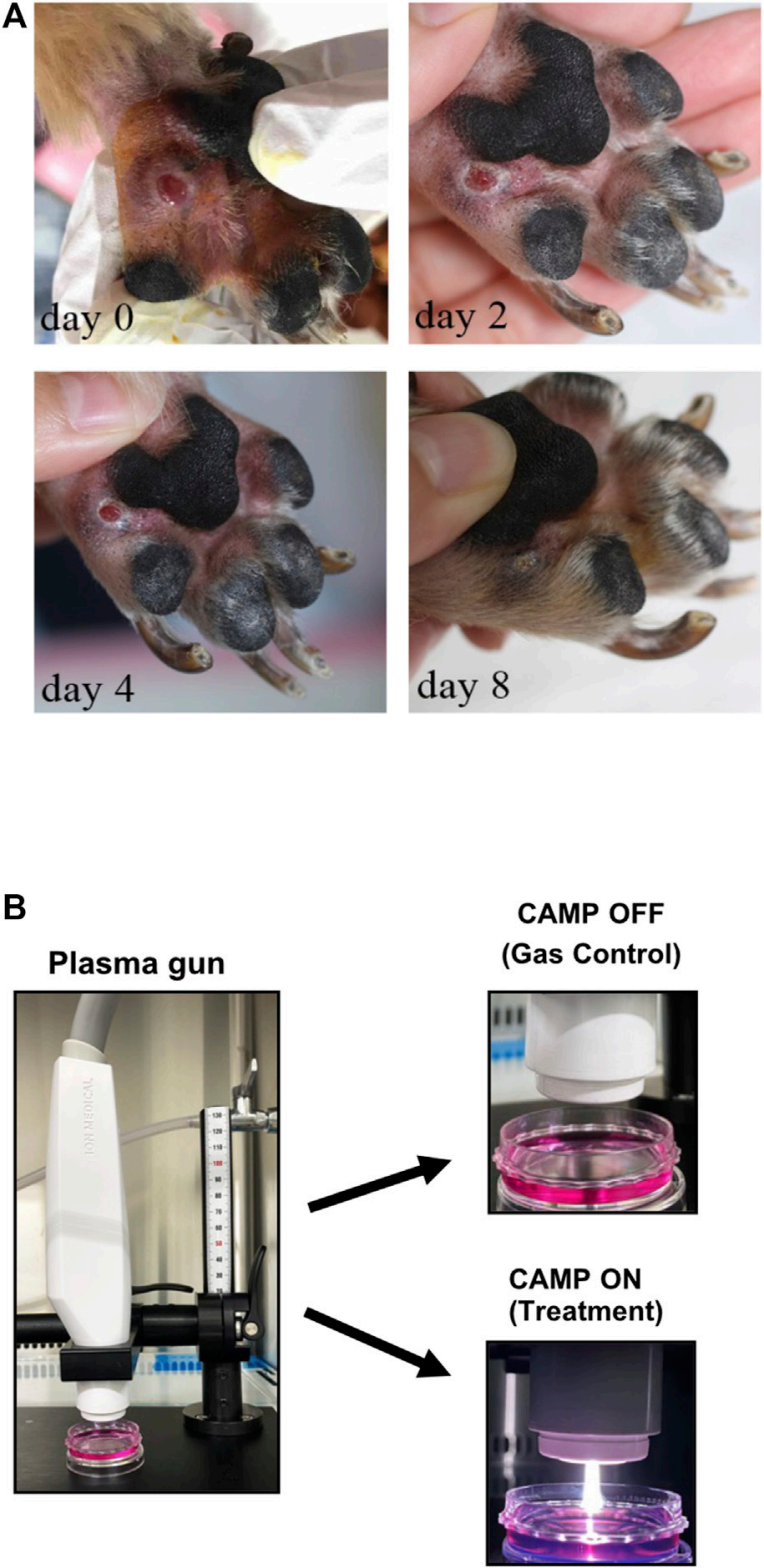
**(A)** Preliminary data of CAMP affecting the acceleration rate of wound healing in a 7-year-old Yorkshire Terrier. CAMP was adjusted to 30 W, and the flow rate of the argon gas was 15 L/min. CAMP was conducted on days 0, 2, 4, and 8. Each day, the procedure was applied to the lesion for 3 minutes. **(B)** Pictures of the CAMP device used in this study.

The CAMP-generating device, from Ion Medical Inc. (Bio Stimulation Microwave Plasma v1.0, Gyeonggi-do, South Korea), was used in this study (Figure 1B). The plasma was generated through microwave energy application (30–50 W; 2,450 MHz) with non-ionized argon, used as a carrier gas. After the gas flowed through an electric field, CAMP was ignited inside the nozzle and emitted outside. The intensity of CAMP could be controlled by altering the power consumption and gas velocity. The flow rate of the argon gas could be adjusted to 10–20 L/min. The distance between the CAMP device and the apex point of the cell culture media was 2 cm. The temperature of the surface contact zone was maintained at <40°C.

### 2.2 Animal

A Yorkshire Terrier, male, 7-year-old dog was referred to SNU-VMTH (Seoul National University Veterinary Medical Teaching Hospital, Seoul, South Korea) with lameness and footpad ulceration of the left forepaw for 4 weeks. History-taking showed no improvement with prednisolone and cephalexin prescribed by a referring veterinarian. This patient was exposed to CAMP, as described in the figure legend.

### 2.3 Cell culture

The immortalized human keratinocyte cell line (HaCaT) was bought from Cell Line Service (Eppelheim, Germany), and canine progenitor epidermal keratinocytes (CPEK) were bought from CELLnTEC (Bern, Switzerland). For the CPEK cell line, 9–20 passages were used, and there was no sign of senescence of cells throughout the experiment period. Both cell lines were grown in Dulbecco’s modified Eagle’s medium (Gibco, Grand Island, NY, United States) supplemented with 10% fetal bovine serum (Gibco, Thermo Fisher Scientific, MA, United States), 100 U/mL penicillin, and 100 mg/mL streptomycin (Gibco) at 37°C and 5% CO_2_ in an incubator with a humidified atmosphere.

### 2.4 Cell viability assay

HaCaT cells were seeded in a 6-well plate at a density of 6.5 × 10^5^ cells/well and further grown for 18 h. Two evaluated methods, CAMP-activated media (CAM) and direct CAMP treatment (dCAMP), were used to determine cell viability. For CAM, media were exposed to CAMP for indicated times and then transferred to the HaCaT cells. Gas-activated media (GAM) were used as a control. After 24 h and 48 h incubation, the number of viable cells in proliferation and/or cytotoxicity was determined. For the direct CAMP treatment, HaCaT and CPEK cells in six-well plates were directly treated with CAMP or gas only. After treatment for 15 min, culture media were substituted with fresh complete media. The cell viability was evaluated using CellTiter 96^®^ AQueous One Solution Cell Proliferation Assay Kit (Promega, Madison, WI, United States), according to the manufacturer’s protocol. Briefly, 200 μL of collected media was added into the 96-well plate after adding one solution and further incubating cells for 2 h. Absorbance at 492 nm was used to detect cell proliferation.

### 2.5 Scratch assay

HaCaT or CPEK cells were seeded at a density of 2.5 $\times 10^{6}$ cells/well in six-well plates and grown for 18 h. After treatment with the CAM or dCAMP regimen, as well as those controls, scratches were developed using a sterile pipette tip. Cells were washed twice using PBS to remove cell debris, and new fresh complete media were added. After 24 h and 48 h of incubation, the area of the wound lesion was observed using a light microscope (Nikon Ti-U; Nikon Instruments, Tokyo, Japan). The wound area was quantified using ImageJ software (Version 1.53k, National Institutes of Health, Bethesda, MD, United States) with the wound-healing tool add-on (Suarez-Arnedo et al., 2020).

### 2.6 Transwell migration assay

The CPEK cells were seeded at a density of 1 $\times 10^{6}$ cells/well in a six-well plate and grown for 18 h. Cells were then treated with 30-s dCAMP or gas. After cell incubation for 15 min, the culture media were replaced by new fresh complete media. After 24 h of incubation, cells were trypsinized and seeded into a 24-well transwell plate (37224, SPLInsert™ Hanging, SPL Life Sciences, Gyeonggi-do, South Korea) and further incubated for 24 h. Then, cells were washed with ×1 PBS twice and fixed with 4% PFA (Biosesang, Gyeonggi-do, South Korea) for 15 min. Later, the cells were stained using 1% crystal violet (61135, Sigma Aldrich, MO, United States) in 2% ethanol for 15 min, and the membrane was rinsed with 1X PBS thrice. The images of cells on the membrane of transwells were captured using a light microscope, and the number of cells per field was counted using ImageJ software.

### 2.7 RNA isolation, library construction, and mRNA sequencing

CPEK cells were seeded at a density of 2.5 $\times 10^{6}$ cells/well in a 6-cm cell culture dish. After incubation for 18 h, cells were directly treated with CAMP or gas for 30 s and then incubated for 15 min before replacing the media with a new fresh complete medium. After media replacement, the cells were further incubated for 24 h. Total RNA from CPEK cells was extracted using the RNeasy Mini Kit (QIAGEN, Hilden, Germany), following the manufacturer’s protocol. RNA-Seq was conducted by Macrogen (Seoul, South Korea). Briefly, RNA-Seq libraries were created using a TruSeq Stranded mRNA Library Prep Kit (San Diego, CA, United States) for the Illumina platform, according to the manufacturer’s instructions. After sequencing, the quality of reads was evaluated, and to decrease the bias of the analysis result, preprocessing was conducted to remove artifacts, including adapter sequence, contaminant DNA, and PCR duplicates using FastQC software (version 0.11.7) and Trimmomatic program (version 0.38). After preprocessing, read data were mapped to the reference genome (CanFam3.1) using the HISAT2 program (version 2.1.0), and aligned reads were generated. Using the reference-based aligned read information, transcript assembly was conducted using the StringTie program (version 2.1.3b). The expression profile values were obtained for each sample for the known transcript, and read count, FPKM (fragment per kilobase of transcript per million mapped reads), and transcripts per kilobase million values were summarized according to the transcript/gene. Using this value, differentially expressed gene (DEG) analysis was conducted using DESeq2, and significant results were selected as |fc| ≥ 2 and nbinomWaldTest raw *p*-value <0.05. Gene Ontology (GO) enrichment was evaluated based on the g:Profiler tool (https://biit.cs.ut.ee/gprofiler/) for a significant list of enriched genes, and gene set enrichment analysis was conducted based on the KEGG (Kyoto Encyclopedia of Genes and Genomes) database (http://www.genome.jp/kegg/) for a significant gene list. To control the false-positive rate, Benjamini and Hochberg’s approach was used to adjust the *p*-values, and a *p*-value <0.05 was considered a significant difference.

### 2.8 Quantitative reverse transcriptase polymerase chain reaction (qRT-PCR)

CPEK cells were seeded at a density of 2.5 $\times 10^{6}$ cells/dish in 6-cm culture dishes and grown for 18 h. Cells were then directly treated with CAMP or gas, and the culture media were replaced with new fresh media after 15 min of incubation. Following further incubation for 24 h, the cells were collected for analysis. Total RNA was extracted using the TRIzol™ reagent (Thermo Fisher Scientific, MA, United States) following the company’s protocol. Total RNA (1 μg) was reverse-transcribed into cDNA using a Verso cDNA synthesis kit (AB1453B, Thermo Fisher, IL, United States) using a MiniAmp Plus Thermal Cycler (Applied Biosystems, Waltham, MA, United States). The primers used for qRT-PCR are listed in the table.

**Table:** 

Target gene	Primer	
	Forward (5’→3′)	Reverse (5’→3′)
COL1A1	CAA GAT GTG CCA CTC CGA CT	AGT GGG GTA TAC GCA GGT CT
COL1A2	GGA TAC GCG GAC TTT GTT GC	CCT TTC TCC ACG TGG TCC TC
COL3A1	TTG CTT CTC GCT CTG CTT CA	CAG ACG CAT ATT TGG CAC GG
COL4A1	GCG TGT GGC TGC TGC T	CAC TTC CCA CAT CCA GAG CC
COL4A2	GAC CGG GAC AAA TAC AGG GG	GAG CTC CAA CTG GAC CCA TC
IL18	ACA CCA GTT GCG ATC CCT G	CGG ACA TCA GGA AAG GAG GTC
IL12a	ATG AGA GTT GCC TGG CTT CC	GAT GCT GCT AAG GCA CAG GA
TLR4	GGC AGC AGG TGG AAC TGT AT	TCC ACG GTT TAC CAT CCA GC
FERMT2	GTG CAC TAA CAA GCA CGA CTG	GTT TTG TTC CCG ACT TGC CC
SPDYA	TCT AAA CGC CCC AAA GGA CC	TAC AGC AGC AGT CCA TCC AC
DSG4	GCA CTC AAC CCC TGT GAG AA	TGG CAG GAT TTC CTG TGT CC
Vimentin	TGA AGC TTG CCC AGA TTC GG	CTT GCG CTC CTG AAA AAC TGC
CDH1	CGG TCT ACC TAG GAC AGC CT	GTT GTA GAG GCC GCT TGA CT
SNAI1	GAA CCT TCT CCC GCA TGT CC	GAG GCT ACA ATG GGG CAA GG
SNAI2	CTG GAC ACG CAC ACA GTG AT	GGA ATG GAG CTG CCG TAG TC
YAP1	ATG CCA TTC CTT TTG CCC AGT	GGC TTG GCA GAC CAG TAA GTC
TAZ	AAC AAG TCG GCT ATG TGG GG	CAG GGA GCT GGA CAA GAC AG
TEAD4	GTG TCC TCT TCT CGT CAA AAA GC	CGG GCC CCA CAT GTA GTG AA
CTGF	TCA GGC CTT GTG AAG CTG AC	ACG GCC ATC TGT GCA TAC TC
β-Actin	GTG GCC GAG GAC TTT GAT TG	GCA ATT CTC TTT CCC TCC CCT

mRNA expression was measured using qRT-PCR. SYBR Green reagents (PowerUp SYBR Green Master Mix, A25741, Applied Biosystems, Thermo Scientific) in a QuantStudio 1 Real-Time PCR System (Applied Biosystems, Marsiling Industrial Estate, Singapore) were used. The mRNA level of each gene was normalized to that of β-actin (housekeeping gene). The relative gene expression was calculated using the comparative Ct method (ΔΔCT Method).

### 2.9 Immunoblotting analysis

Cells were seeded at a density of 2.5 $\times 10^{6}$ cells/dish in 6-cm culture dishes and grown for 18 h. Cells were then treated with dCAMP or gas. After media replacement and further incubation for 24 h, cells were washed twice in ice-cold PBS and lysed with RIPA buffer containing proteinase inhibitors. The concentration of proteins was quantified using the Pierce™ BCA protein assay kit (Thermo Scientific, Abbott Park, IL, United States). Afterward, 80 µg of proteins was separated using 10% sodium dodecyl sulfate-polyacrylamide gel electrophoresis (SDS-PAGE) and transferred to a PVDF membrane. The membrane was blocked using 5% BSA for 1 hour at RT, and specific antibodies were incubated at 4°C overnight. The primary antibodies used (at a dilution of 1:1000) included rabbit anti-E-cadherin (24E10, #3195S, Cell Signaling, MA, United States), rabbit anti-Snail (C15D3, #3879S, Cell Signaling), rabbit anti-Slug (C19G7, #9585P, Cell Signaling), rabbit anti-vimentin (#A11952, ABclonal, MA, United States), and rabbit anti-YAP/TAZ (D24F4, #8418S, Cell Signaling). The blotted membrane was subsequently incubated with HRP-conjugated anti-GAPDH (G9, #sc-365062, Santa Cruz, TX, United States) used as the internal control. After washing using 1% TBST and blotting using goat anti-rabbit, the membrane was incubated with HRP-conjugated IgG secondary antibody (1:5000 dilution, #31460, Thermo Scientific) in 5% skim milk. Membranes were developed using the ECL substrate (Pierce™ ECL Western Blotting Substrate, 32106, Thermo Scientific) and visualized using Alliance Q9 Mini (Cambridge, United Kingdom) and quantified using ImageJ software.

### 2.10 Statistical analysis

Data are expressed as mean ± SD or SEM with three independent experiments. Statistical analyses were conducted using Student’s t-test and one-way ANOVA followed by *post hoc* Dunnett’s test using GraphPad Prism 9 software (version 9.5.0). All comparisons were made relative to its control, and significant differences have been presented as *p < 0.05, **p < 0.01, and ***p < 0.001.

## 3 Results

### 3.1 Direct CAMP treatment shows a promising effect on activating the wound-healing process in human keratinocytes (HaCaT)

**FIGURE 2:**
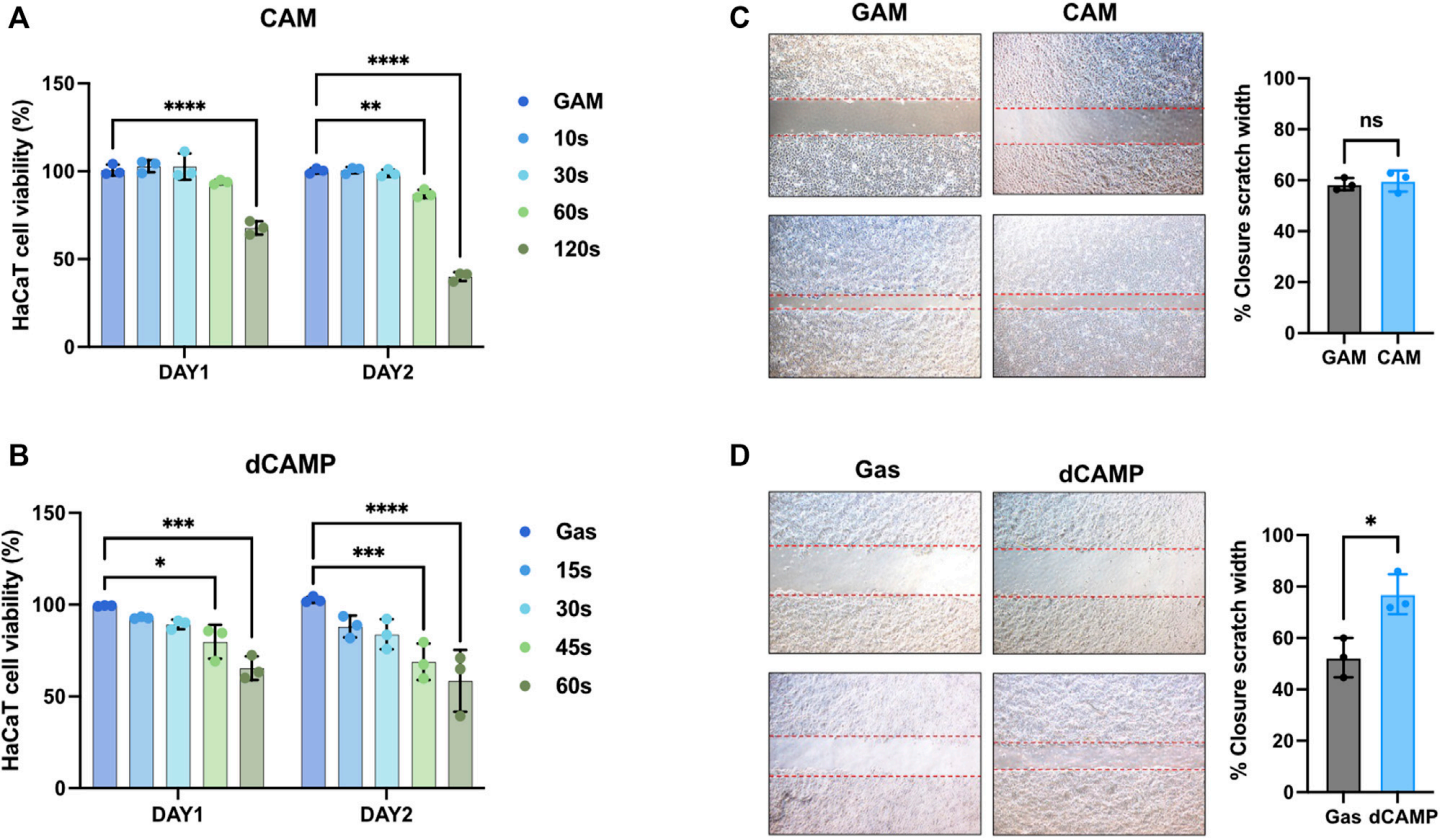
Direct CAMP treatment (dCAMP) shows promising effects on activating the wound-healing process in human keratinocytes. **(A,B)** Cell viability of HaCaT cells treated with CAMP-activated media (CAM) and direct CAMP (dCAMP). For wound-healing assay, the scratches were created using pipetted tips. HaCaT cells were treated with 30-s CAM **(C)** and 30-s dCAMP **(D)**. The wound areas were measured at 0 h and 24 h, and the adjacent graph shows the quantitative data on the closure area. Data are presented as mean ± standard error, *p < 0.05, compared with the gas treatment group.

The Yorkshire Terrier dog (male, 7-year-old) was referred to Seoul National University Veterinary Medical Teaching Hospital with lameness and footpad ulceration of the left forepaw for 4 weeks. The physical examination and blood analysis showed no specific finding. During the dermatological examination, the dog suffered edema, ulceration, and moderate erythema on his left forepaw footpad. Cytology showed pyogranulomatous inflammation with cocci, suggesting a foreign body rather than neoplasia. However, no foreign body with a puncture was found, and the lesion was extruded. During treatment using CAMP, discomfort, edema, erythema, and size of the lesion were reduced, and the lesion was resolved on day 8 (Figure 1A). To investigate the appropriate treatment to further experiment, the treatment techniques were set using both CAMP-activated media (CAM) and direct CAMP treatment (dCAMP) (Figure 1B). For the CAM-treated experiment, the HaCaT cells were treated with CAM, the culture media derived from the CAMP-treated medium with various exposure times, and further incubated. At 24 h and 48 h, cell viability was analyzed. The results showed that cells treated with 120-s CAM showed a significant decrease in cell viability (67.79% ± 3.80%), compared with gas-activated media (GAM), while the other doses showed no significant difference at 24 h (Figure 2A). At 48 h time point, cell survival results showed a significant decrease in cell survival in 60- and 120-s CAM-treated HaCaT cells (87.15% ± 2.34% and 40.08% ± 2.49%, respectively), compared with GAM (Figure 2A). Therefore, 60 s CAM was selected for further experiments. However, in a scratch assay, the results exhibited no significant effect of CAM on wound-healing activity (Figure 2C). In case of dCAMP, HaCaT cells were directly treated with CAMP for the indicated exposure times and then further incubated with new fresh complete media for 24 h and 48 h. Cell viability assay results revealed that the percentage of cell proliferation in treatment with dCAMP for 45- and 60 s at 24 h and treatment with dCAMP for 30 s, 45 s, and 60 s at 48 h (for 24 h, 79.81% ± 9.21% and 65.27% ± 6.42%, respectively; for 48 h, 83.84% ± 8.18%, 68.92% ± 9.95%, and 58.38% ± 16.82%, respectively) shows a significant reduction rate in cell proliferation (Figure 2B). According to ISO 10993-5:2009, percentages of cell viability above 80% are considered non-cytotoxic (Iso, 2009); therefore, 30 s of dCAMP was selected for use in the wound-healing assay. Although there was no significant effect of CAM on wound-healing activity, the results of the dCAMP treatment method illustrated that directly treated HaCaT cells with CAMP significantly increased the percentage of wound closure width compared with gas control (77.03% ± 7.73% vs. 52.37% ± 7.66%, Figure 2D). All these results suggested that the dCAMP treatment regimen exhibits a favorable effect on wound healing in HaCaT cells.

### 3.2 Direct CAMP treatment showed preferable activities in promoting cell migration and cell invasion abilities of wound healing in canine keratinocytes (CPEK)

**FIGURE 3:**
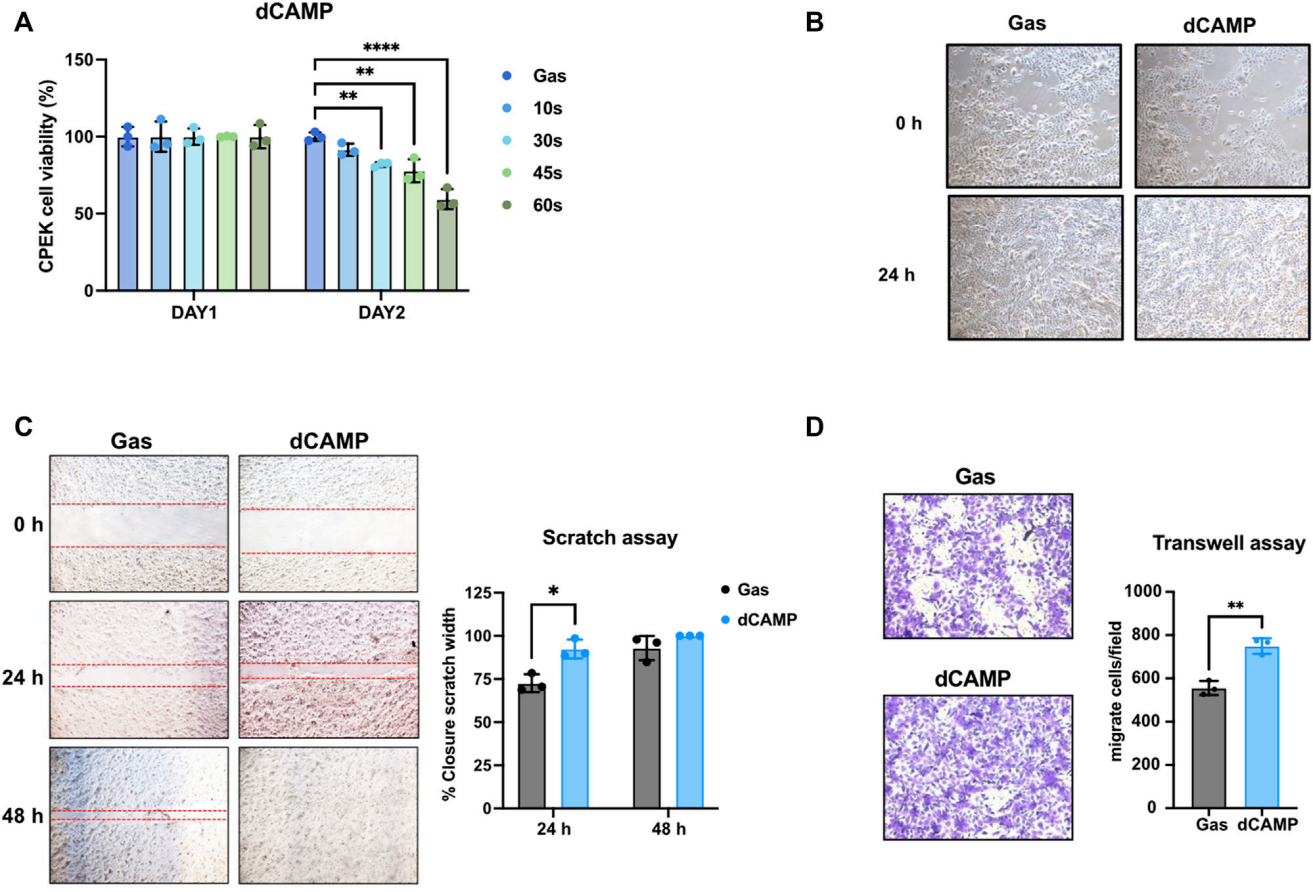
dCAMP treatment showed preferable activities to augment cell migration and invasion abilities in canine keratinocytes’ wound healing. **(A)** Cell viability of CPEK cells at 24 h and 48 h after being directly treated with various exposure times of CAMP or gas. **(B)** Cell morphology of CPEK cell before and after treatment with 30-s dCAMP. **(C)** Scratch assay shows the significantly higher wound closure rate in 30-s dCAMP-treated CPEK cells, than gas. **(D)** Transwell assay shows the positive effects of 30-s dCAMP on the wound-healing process by promoting CPEK cell migration and invasion. Data are presented as mean ± standard error, *p < 0.05, **p < 0.01, and ****p < 0.001 compared with the gas-treated group.

Since our data suggested that dCAMP might provide a positive effect on wound healing in dogs (Figure 1A), the molecular mechanisms of those effects on dog keratinocytes were evaluated using the CPEK cell line. As shown in Figure 3A, after treating cells with dCAMP for various exposure times, cell viability assay showed no significant effect on cell survival with all those times of treatment at 24 h after incubation; however, the proliferation rate of dCAMP-treated CPEK cells began to significantly reduce at 48 h after treatment for 30 s, 45 s, and 60 s, compared with gas (80.95% ± 4.22%, 74.48% ± 1.20%, and 59.40% ± 6.51%, respectively). Therefore, dCAMP treatment for 30 s exposure time was selected for further study due to the percentage of cell survival being higher than 80. In addition, there was no cell morphology change before and/or after dCAMP treatment for 30 s exposure time, as shown in Figure 3B.

To evaluate the wound-healing activity, scratch assay and transwell assay were performed. For the scratch assay (Figure 3C), dCAMP-treated cells show a significantly higher acceleration rate of lesion closure than gas control at 24 h (92.32% ± 5.13% vs 72.61% ± 5.12%). Unfortunately, there was no difference between both groups at 48 h; the reason was that the scratch area of the dCAMP-treated cells had been utterly closed before the evaluation time-point (100.00% vs 92.92%). For the transwell assay (Figure 3D), dCAMP-treated CPEK cells demonstrated a significantly higher number of migratory and invasive cells than those treated with gas (749.33 ± 35.92 cells/field vs 556.00 ± 32.42 cells/field). These suggested that dCAMP treatment promotes cell migration and cell invasion of canine keratinocytes.

### 3.3 RNA sequencing analysis of dCAMP-treated CPEK cells

**FIGURE 4:**
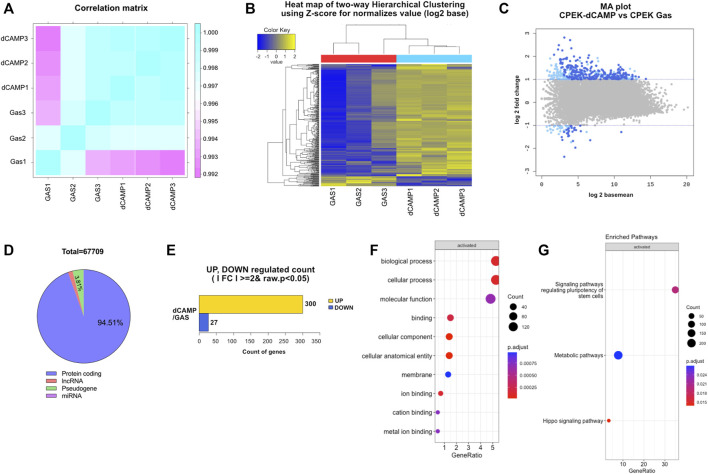
RNA sequencing analysis of 30-s dCAMP-treated CPEK cells compared with gas-treated cells. **(A)** Correlation matrix presents correlation coefficients between dCAMP-treated and gas-treated cells. The closer the correlation coefficient value is to 1, the higher the similarity between samples. **(B)** Two-dimensional hierarchical cluster analysis of differentially expressed genes between dCAMP-treated and gas-treated groups; an increase in expression is shown in yellow hues, and reductions are in blue hues. **(C)** MA plot shows the relationship between the significance of the differential expression test (log_2_ fold-change) and mean-normalized expression between dCAMP treatment and gas treatment. Gray represents no significant change, light blue represents absolute log FC ≥ 2, and dark blue represents absolute log FC ≥ 2 and *p*-value <0.05. **(D)** Total number of genes regulated by direct CAMP treatment. **(E)** Number of genes significantly up- and downregulated by CAMP-treated cells. The GO categories for **(F)** biological process and **(G)** enriched pathway of KEGG.

RNA-Seq data were generated from CPEK cells directly treated with CAMP for 30 s. To ensure comparability between the gene expression profiles of dCAMP-treated cells and gas control, a correlation heatmap was generated (Figure 4A). The correlation coefficient graphical diagram showed a positive correlation between both groups. The cluster analysis, as presented in Figure 4B, was performed and grouped based on the degree of similarity of the expression pattern for each gene in each sample, using FPKM values of each gene. From the MA plot (Figure 4C), genes with at least a two-fold difference and adjusted *p* < 0.05 were selected to determine DEGs between the two groups. Among those 67,709 genes (protein-coding 63,959 genes, pseudogene 2,576 genes, lncRNA 1,096 genes, and miRNA 42 genes), 327 significantly regulated genes were identified; 300 genes were upregulated; and 27 genes were downregulated in the dCAMP-treated CPEK cells (Figures 4D, E). From DEGs (Supplementary Data), the GO analysis result (Figure 4F) revealed the role of dCAMP treatment in the biological process and molecular function. Subsequently, KEGG analysis showed the associated pathway induced by direct CAMP treatment; including signaling pathways regulating pluripotency of stem cells, metabolic pathway, and Hippo signaling pathway (Figure 4G).

### 3.4 Direct CAMP treatment upregulated target genes involved in wound healing

**FIGURE 5:**
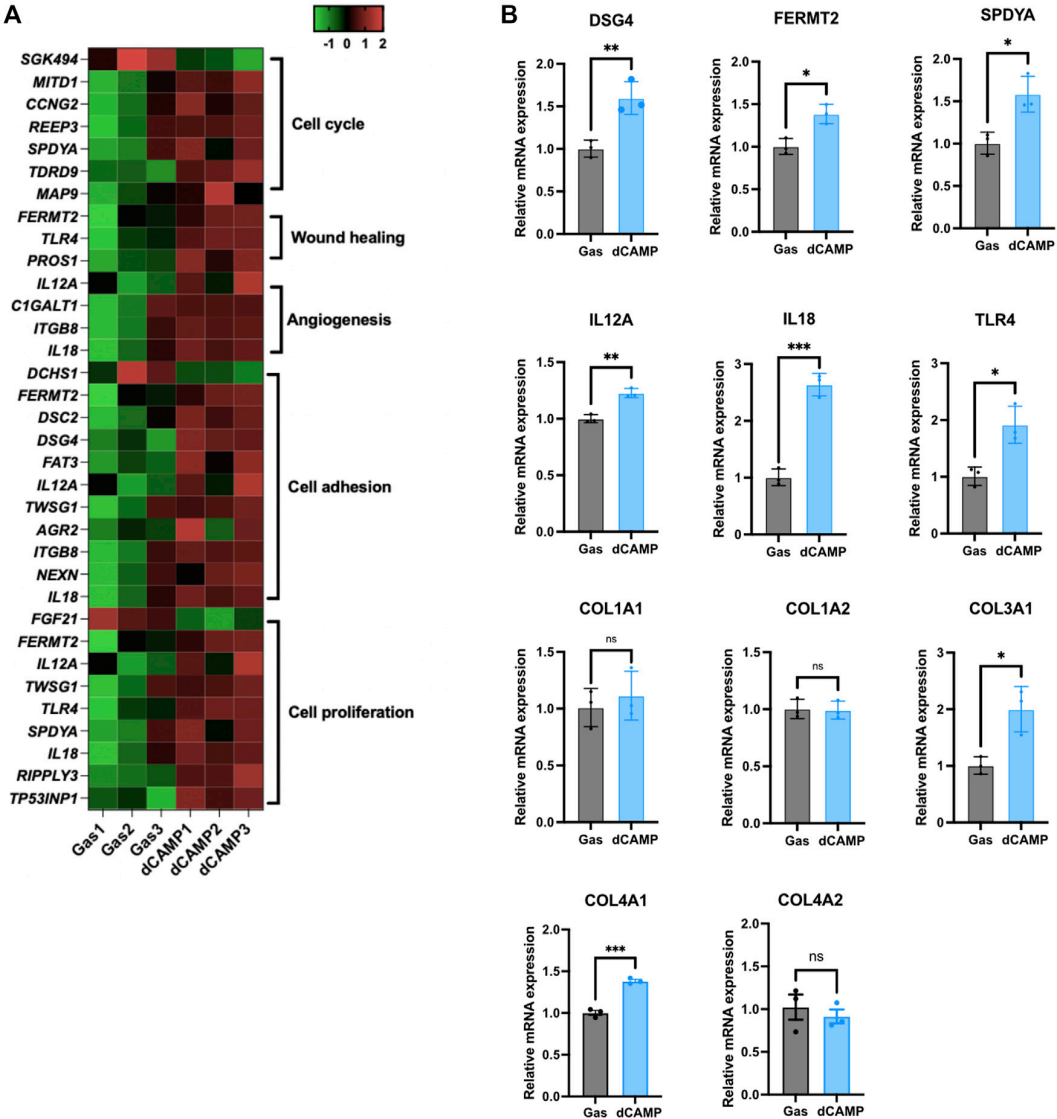
dCAMP treatment upregulated target genes associated with wound healing in CPEK cells. **(A)** Heatmap represents the gene regulated by dCAMP in various pathways; raw data was obtained from GO analysis. **(B)** mRNA expression analysis by qRT-PCR shows that dCAMP regulated expression of various genes in the wound-healing process, consistent with the enrichment analysis results. Data are presented as mean ± standard error, *p < 0.05, **p < 0.01, and ***p < 0.001 compared with the gas-treated group.

To verify the gene enrichment analysis data from RNA-Seq, shown in the heatmap (Figure 5A), we selected several significantly regulated genes involved in the cell cycle, cell proliferation, angiogenesis, cell adhesion, and wound healing. The selected genes, including DSG4, FERMT2, SPDYA, IL12a, IL18, and TLR4*,* were significantly upregulated by dCAMP treatment, consistent with the heatmap data. Since collagen genes are involved in the wound-healing process, we examined the expression of collagen genes. Our data demonstrated that there was no change in Col1A1 and Col1A2 mRNA expression; however, Col3A1 mRNA levels were significantly upregulated in dCAMP-treated CPEK cells compared with the control. For the gene expression of collagen IV, which is abundant in the basement membrane, Col4A1 was upregulated in dCAMP-treated cells, but not for Col4A2, compared with the gas control (Figure 5B).

### 3.5 Direct CAMP treatment plays roles in Hippo and EMT pathways involved in wound healing

**FIGURE 6:**
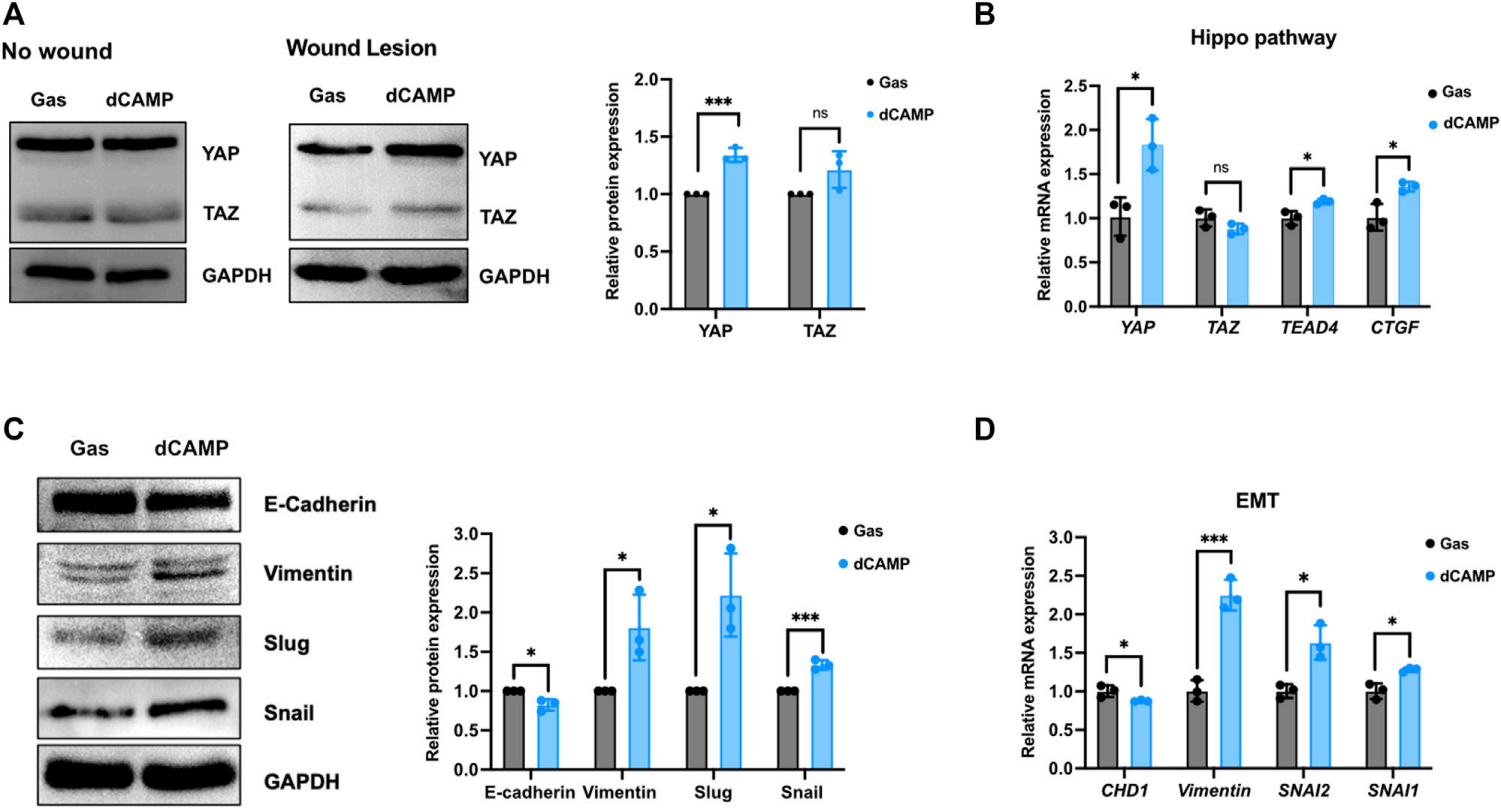
Direct CAMP treatment plays roles in EMT and Hippo pathway contributing to promoting wound-healing activity in canine keratinocytes. **(A)** Downstream targets of the Hippo pathway, YAP and TAZ protein expression, under normal conditions (no wound) and wound conditions (wound lesions) after treatment with 30-s dCAMP or gas. The adjacent graph presents the quantitative expression of the relative proteins. **(B)** qRT-PCR data verified the mRNA gene expression in the Hippo pathway. Immunoblotting **(C)** and qRT-PCR **(D)** results show that 30-s dCAMP improved cell migration by inducing EMT markers. Data are presented as mean ± standard error, *p < 0.05, **p < 0.01, and ***p < 0.001 compared with the gas-treated group.

A number of studies have reported that the Hippo pathway plays a role in skin homeostasis and wound-healing process (Wang et al., 2017; Rognoni and Walko, 2019). As shown in Figure 6A, the Western blotting data showed no induction of YAP or TAZ protein after treatment with 30-s dCAMP in CPEK cells without wound lesions. Interestingly, with wound lesions, the results showed significant induction of YAP protein (1.4-fold), but not TAZ protein, which is specific to dCAMP-treated CPEK cells. Subsequently, the mRNA expression showed substantial upregulation in YAP, TEAD4, and its downstream target CTGF/CCN2 mRNA level (Figure 6B). In addition, EMT is a critical process to promote cell migration, which is essential for the wound-healing process. Therefore, EMT markers were evaluated at both the transcription and translation levels. CPEK cells with wound lesions treated with dCAMP for 30 s were used for the investigation. The data revealed the protein downregulation of E-cadherin (0.8-fold) and protein upregulation of vimentin (1.8-fold), Slug (2.3-fold), and Snail (1.4-fold) (Figure 6C). These were confirmed using qRT-PCR to measure mRNA expression levels. The data showed the same pattern as protein-level expression: downregulation of CHD1 and upregulation of vimentin, SNAI1, and SNAI2 mRNA expression (Figure 6D). These results suggested that dCAMP treatment plays roles in different mechanisms involved in wound healing, including the Hippo pathway and EMT process.

## 4 Discussion

Impaired wound healing is the unsolved process of resolving the injured tissues due to improper wound care or infections. The more accelerated the time to heal, the better the resolution of the curative lesions (Guo and Dipietro, 2010). The epidermal wound healing is a major consideration since the epidermis finally covers all the wound lesions, contributing to wound closure in the wound-healing process. The stratified epithelium is made of keratinocytes organized in the epidermis layer, which can proliferate and endanger daughter cells that migrate to the skin surface in supra-basal layers (Rittié, 2016). Many studies on human models have confirmed the benefit of plasma treatment. For instance, human diabetic foot ulcer wounds were subjected to a study, and the results showed that plasma therapy affects wound surface reduction and wound repair time independent of the infection background (Stratmann et al., 2020). In the full-thickness skin wound model in rats, plasma treatment could promote skin wound healing by accelerating cell contraction, re-epithelialization, neovascularization, and inflammatory response but did not change collagen bundles (Zhang et al., 2019). However, there is not much information on using canine models for plasma treatment. Our study indicates the potential of the direct-CAMP regimen, which significantly promotes the rate of wound repair in canine keratinocytes. In addition, the effects of dCAMP treatment in cell migration and invasion assay were confirmed, and the results showed a positive outcome that direct treatment with CAMP contributes to favorable effects on canine wound repairing.

**FIGURE 7:**
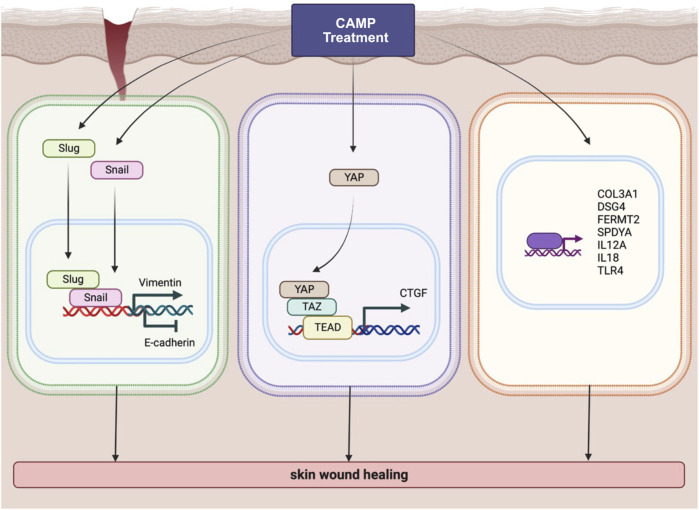
Schematic diagram represents various pathways involved in wound-healing activity induced by CAMP treatment.

After reassuring about the effect of dCAMP treatment on wound healing, the RNA-Seq was conducted in our study to identify the potential molecular mechanisms and pathways that contribute to lesion repair. The GO analysis results exhibited the notable regulated genes involved in wound healing (Figure 5A). To confirm the expression of those regulated genes, qRT-PCR was conducted with selected representative target genes in the wound healing-associated biological process. The expression of transmembrane cadherin proteins, desmogleins (DSGs), which promote epidermal integrity by inducing epithelial cell adhesion (Johnson et al., 2014), was increased after direct treatment with CAMP to canine keratinocytes. Fermitin family homologs (kindlin) are intracellular adapter proteins that play roles in integrin-containing focal adhesions, the regulatory effect between actin microfilaments and extracellular matrix (ECM) (Meves et al., 2009). A previous report suggested that cell motility and formation of cell–cell contacts were particularly affected by kindlin-2, FERMT2, in human keratinocyte cell lines (He et al., 2011). Our results also showed that direct treatment with CAMP affects FERMT2 upregulation in CPEK cells. Additionally, SPDYA (Speedy/RINGO cell cycle regulator family member A) is a CDK2 activator and directly binds and promotes the degradation of the CDK inhibitor, p27^Kip1^ (Porter et al., 2003; McAndrew et al., 2007). *SPDYA* gene was also upregulated in dCAMP-treated cells. The other genes, including IL12a, IL18, and TLR4, the crucial molecules in non-infectious inflammation, which play essential positive roles in early wound repair (Kämpfer et al., 1999; Chen et al., 2013; Li et al., 2015), were also affected by the dCAMP regimen (Figure 7; right). In addition, collagen plays important roles in wound healing, including inflammation, angiogenesis, and extracellular matrix (ECM) remodeling (Mathew-Steiner et al., 2021). It is suggested that collagen III is the first to be synthesized in the early stages of wound healing and is substituted by dominant skin collagen I (Mathew-Steiner et al., 2021). Among those collagen subtypes, collagen IV was abundantly found in the basal membrane of skin, and it is a major component at the dermal–epidermal junction, where it is mostly found in the lamina densa (Abreu-Velez and Howard, 2012). In this study, we found that the collagen gene expression, measured by using qRT-PCR, was regulated by dCAMP treatment. Collagen III, Col3A1, was significantly increased in the dCAMP-treated group compared with the gas control, whereas there was no change in collagen I, Col1A1, and Col1A2 gene expression. In addition, direct treatment with CAMP also regulated the collagen IV gene expression: upregulated Col4A1 mRNA level and no effect on Col4A2 expression in canine keratinocytes.

According to the data from the KEGG pathway analysis, there are top three remarkable pathways: signaling pathways regulating pluripotency of stem cells, metabolic pathway, and Hippo signaling pathway. Since the study reported that in full-thickness skin wounds, siRNA-mediated knockdown of YAP/TAZ, coactivators of the Hippo pathway, notably impeded the rate of wound closure and reduced the transforming growth factor-β1 (TGF-β1) expression in the wound by altering CTGF/CCN2, Smad-2, p21, and Smad-7 expression (Lee et al., 2014). In addition, YAP/TAZ is a necessary protein that maintains skin homeostasis, as the deletion of Yap1/Wwtr1 shows a delayed proliferation of basal layer cells and contributes to hair loss and tissue damage (Elbediwy et al., 2016). In particular, YAP functions as a molecular switch of stem/progenitor cell activation in the epidermis. YAP shows the ability to increase the epidermal stem/progenitor cell proliferation through regions in its N-terminus, including the TEAD-binding domain, whereas its C-terminus, including the YAP transactivation domain, plays a role in controlling the balance between proliferation and differentiation in the interfollicular epidermis (Beverdam et al., 2013). Therefore, we further determined the protein expression of YAP and TAZ after directly treating canine keratinocytes with CAMP (Figure Figure7; middle). Our data showed that under normal conditions, treatment with CAMP did not change the YAP/TAZ protein expression. In contrast, the YAP protein significantly increased in dCAMP-treated cells under wound conditions. This indicated that direct CAMP enhanced the Hippo signaling pathway by increasing YAP protein upon cell damage. Furthermore, there are studies indicating that the Hippo signaling pathway is also involved in the epithelial–mesenchymal transition (EMT) process (Park et al., 2019; Cheng et al., 2020). Here, the biomarkers of the EMT process, including vimentin, Snail, Slug, and E-cadherin were confirmed using immunoblotting and PCR. The results showed a preferable effect on the transition from epithelial-like to mesenchymal-like cells, which implies that the direct treatment with CAMP promotes the re-epithelialization process in canine keratinocytes’ wound healing by inducing EMT (Figure 7; left).

Unfortunately, there were a limited number of actual dog participants due to the number of patients in the animal hospital and the wound types and stages are largely different. Even though we used the keratinocyte cell line, which resembles the monolayer of the epidermis, in this research, the animal model is still required. Moreover, in each layer of skin, the mechanistic pathway of wound healing and their biological molecules cannot completely be discrete. Therefore, the co-culture of different skin cell lines may provide molecular mechanistic information in depth in future study. For the CAMP device, the knowledge about the molecular mechanisms of various signaling pathways, clinical outcomes, and safety profiles was still required.

Overall, this study suggests that CAMP exhibits beneficial outcomes on wound healing in canine keratinocytes through the Hippo pathway, the EMT process, and the genes involved in wound healing, revealed by RNA-Seq and *in vitro* experimental techniques using the canine keratinocyte cell line.

## Data Availability

The data presented in the study are deposited in the GEO database of NCBI, accession number GSE222898.
